# Ordering folate assays is no longer justified for investigation of anemias, in folic acid fortified countries

**DOI:** 10.1186/1756-0500-3-22

**Published:** 2010-01-25

**Authors:** A Majid Shojania, Kenneth von Kuster

**Affiliations:** 1 St Boniface General Hospital, 409 Tache Avenue, Winnipeg, Manitoba, R2H 2A6 Canada

## Abstract

**Background:**

Since 1998, in the countries where there is mandatory fortification of grain products with folic acid, folate deficiency has become very rare. Consequently, we decided to find out whether there is any justification for ordering folate assays for investigation of anemias.

**Methods:**

We reviewed serum folate (SF) and red cell folate (RF) data at two teaching hospitals in Canada. At the Health Sciences Centre (HSC) the folate data for the year 2001 were analyzed and the medical records of those with low SF or low RF were reviewed. At St. Boniface General Hospital(SBGH)all folate data between January 1996 and Dec 31,2004 were analyzed and the medical records of all who had low RF between January 1,1999 and December 31,2004 were reviewed.

**Results:**

In 2001, at HSC, 11 out of 2154(0.5%)SF were low(<7.0 nmol/L) and 4 out of 560 (0.7%) RF were low (<417 nmol/L). In no subject with low SF or RF could the anemia be attributed to folate deficiency. At SBGH during the 3-year-period of 1999-2001, 19 out of 991(1.9%) had low RF (<225 nmol/L) but in only 2 patients (0.2%) the low RF was in folate deficiency anemia range; but neither of them had anemia.

**Conclusion:**

In countries where there is mandatory fortification of grain products with folic acid, folate deficiency to the degree that could cause anemia is extremely rare. Ordering folate assays for investigation of anemias, in these countries, is waste of time and money. The result of these tests is more likely to mislead the physicians than to provide any useful information.

## Background

During the period of 1940-60, folate deficiency in a patient was not recognized until it had advanced enough to cause megaloblastic anemia. With the development of folate assays, folate deficiency could be identified much earlier. These assays provided data which showed the high prevalence of folate deficiency in: pregnancy, premature infants, hemolytic anemias, malabsorption syndrome and alcoholics. Furthermore, research showed a link between folate deficiency and pregnancy complications such as abruptio placenta, eclampsia, congenital malformations, especially neural tube defect (NTD), hyperhomocysteinemia, occlusive cardiovascular disorders and neuropsychiatric disorders. Serum folate (SF) and red cell folate (RF) became commonly ordered tests, for investigation of anemias and conditions cited above.

In 1996, in order to reduce the development of NTD, US Food and Drug Administration mandated that by January 1, 1998 all grain products must be fortified with 0.14 mg of folic acid per 100 g of grain [1]. Health Canada also mandated a similar folic acid fortification of grain products with the deadline of November 1, 1998 [2]. At that time, it was estimated that such fortification of grain products would add an average of 0.1 mg folic acid to the daily intake of folate by adults. This mandatory fortification of grain products markedly reduced the prevalence of folate deficiency and increased the mean SF and RF of the population [3-7].

In February, 2002, one of us (AMS), after noticing that in the year 2001, less than 1% of folate assays performed were low, notified the physicians and the wards at St. Boniface General Hospital (SBGH) that "Since mandatory fortification of grain products with folic acid, folate deficiency has become extremely rare. Consequently, the laboratory would no longer accept an order for folate assay, unless the physician could justify ordering these tests". Subsequently, Health Science Centre (HSC) and all other public laboratories (laboratories located in hospitals) in the province of Manitoba, discontinued providing folate assays and referred the tests to SBGH. SBGH remained the only public laboratory for performance of folate assays in the Province of Manitoba, Canada. This policy resulted in marked reduction of folate assays performed at SBGH for all public laboratories in Manitoba. Consequently, we decided to find out whether there was any need for folate assays for investigation of anemias.

## Methods

This was a retrospective study of folate data and a review of all medical records of the patients with low SF or RF at HSC and SBGH, the two teaching hospitals in Manitoba. According to the policy at the time, the approval of the Medical Director and Director of Health Records of the two institutions was obtained prior to review of patient's records.

SBGH had been using the same L. Casei microbiological assay of serum folate and red cell folate since 1966. SF was determined using the method of Waters and Mollin [8]. RF was determined by measuring SF and whole blood folate and calculating the red cell folate [9]. SBGH data was used to determine the changes in SF and RF before and after folic acid fortification. During 2001, HSC was using a competitive binding assay of folate (IM_X _system, Abbott Laboratories Diagnostic Division, Abbott Park, Il). Normal range for this assay was established in 1997. HSC data was used to demonstrate the prevalence of low SF and low RF post folic acid fortification era.

KvK reviewed all the folate assays performed at HSC in 2001 and reviewed the hospital records of those who had low SF or RF, to find out the cause of low folate and relation of the low folates to the patient's anemia. AMS, using the laboratory information system (LIS), reviewed all folate assays performed at SBGH for the periods 1996-2004 and reviewed the hospital records of only those who had low RF, to see if there was any explanation for low RF or any hematological abnormality to support the diagnosis of folate deficiency anemia (macrocytosis and/or increased number of hypersegmented polymorphs or megaloblastic changes in the bone marrow).

## Results

### Analysis of SF data at HSC

The normal SF at HSC in 2001 was >7.0 nmol/L. Of the 2154 SF assayed in 2001, 11 (0.5%) were low (SF of 3.9-6.6 nmol/L). All 11 patients were anemic but their SF were not as low as those seen in folate deficiency anemia (see comments below). Only one case had a high mean corpuscular volume (MCV). This patient had ethanol induced pancreatitis and an SF of 4.8 nmol/L. Two patients with celiac disease had slightly low serum folates of 6.3 and 6.1 nmol/L. One referred-in sample had a low SF, low serum B_12 _(SB_12_) and low serum ferritin (malabsorption syndrome?). In the other seven patients there was no evidence of folate deficiency anemia and no explanation could be found to why their SFs were low.

### Analysis of RF Data at HSC

The normal range for RF at HSC was >422 nmol/L. In 2001, among 560 RFs performed, 4 patients (0.7%) had low RF. In one case, the low RF of 417 nmol/L was due to B_12 _deficiency. The second case was the same patient with ethanol induced pancreatitis who also had low SF. The other two had celiac disease.

### Analysis of the SF Data at SBGH

The normal range for serum folate at SBGH was >6.8 nmol/L. The percentage of low SF at SBGH was much higher than that in HSC, because SBGH was using a microbiological assay which gives falsely low results if the patient is on antibiotics or antimetabolites. Table 1 shows the number of SF, the percentage of those with low SF and the mean SF in different periods between 1996 and 2004. During the 24-month-period of January 1, 1996-December 31, 1997, 2602 SF were assayed with the mean SF of 17.1 nmol/L. Of these, 598 (22.9%) were below normal. During the 36-month-period of January 1, 1999-December 31, 2001, 4464 SF were performed with the mean SF of 30.1 nmol/L. Of these, 242 (5.2%) were below normal. During the 3-year-period of January 2002-December 2004, when physicians had to explain why a SF or RF was needed (generally because the patient had a macrocytic anemia with normal SB_12 _or because malabsorption syndrome was suspected) only 24 SF were assayed, with the mean SF of 37.9 nmol/L. Significantly, none of these 24 SF were low. Ironically, Physicians, in private office, who did not have to provide reason for ordering folate assays, continued to order folate assays, at increasing rate. During the 3-year-period of 2002-2004 when public laboratories performed only 24 SF, the private laboratories charged Manitoba Health Department for performing 28,136 serum folate assays.

**Table 1:** Serum folates (S. folate) performed at SBGH (1996-2004)

Year	Number	# of low S. folate	% of low S. folate	Mean S. Folate (nmol/L)
1996-8	2602	598	22.9%	17.1

1999-01	4464	242	5.20%	30.1

2002-04	24	0	0.0%	37.9

The difference between the mean, number and percentage of low serum folates for the periods of 1996-98 and 1999-2001 is significant (P < 0.001) but between 1999-2001 and 2002-04 is not significant.

### Analysis of the red cell folates data at SBGH

The normal RF at SBGH was >225 nmol/L. Red cell folates at SBGH were also determined by microbiological assays. However, because the whole blood has to be diluted 1:10 for RF assay, this dilution minimizes the inhibitory effect of most of the antibiotics and antimetabolites on the growth of L. Casei used for folate assay. Because of this, and also because low red cell folate correlates much better than low serum folate with folate deficiency anemia, a more detailed analysis was performed on the RF, and only the medical records of those with low RF were reviewed. Furthermore, because some grain companies had started fortifying their products with folic acid by late 1996 and most of the others by January, 1998, we analyzed the RF data year by year to see the effect of this folic acid fortification. Table 2 shows the number and the percentages of those with low RF for the periods of January 1996-December 2004. The percentage of low RF in 1996 was 18.5%; in 1997, 11.5%; in 1998, 4.5% and for the 3-year period of 1999-2001, it was 1.6%. The mean RF rose from 509 nmol/L in 1996 to 947 nmol/L in the post folic acid fortification period. During the 3-year-period of 1999-2001, 19 of 991 RFs (1.9%) were low. Of these, three had normal SF, 9 patients were ethanol abusers with alcoholic liver disease; 7 patients had celiac disease; two patients had low RF because of a severe B_12_deficiency; and one patient had AIDS with extreme malnutrition, (low RF, SB_12 _and serum ferritin). Of 7 patients with celiac disease, only one had a high MCV of 103.3 fl with RF of 173 nmol/L. Only two patients had RF in the folate deficient anemia range of <100 nmol/L, but were not anemic.

**Table 2:** Red cell folates (R. folate) performed at SBGH (1996-2004)

Year	Number	# of low R. folate	% of low R.folate	Mean R. folate (nmol/L)
1996	511	95	18.5	509

1997	474	56	11.5	588

1998	581	17	4.5	858

1999-01	991	19	1.9	947

2002-04	34	1	2.9	831

The difference between the mean, number or percentage of low red cell folates in 1996 and 1997 is significant (*P *< 0.01), between those of 1997 and 1998 is also significant (*P *< 0.001), but the differences between those of 1998, 1999-2001 and 2002-2004 are not significant, based on Chi square calculation and t-test(By January first 1998 almost all of grain products in Canada were fortified with folic acid).

During the 3-year period of 2002-2004, when physicians had to justify why folate assay was needed, only 34 RF were assayed with the mean RF of 831 nmol/L. Of these 34 patients only one patient with celiac disease, had a slightly low RF of 206 nmol/L with a normal SF of 7.3 nmol/L.

Limitation of this study - Because this was a retrospective study, we could not make sure that proper data is available for investigation of the anemia in every patient. Folate assays were not necessary ordered at the same time that complete blood count (CBC) was ordered; and not all patients had proper description of blood smear or additional confirmatory tests such as serum homocysteine or bone marrow examination. We used the closest CBC result prior to ordering folate assays.

## Discussion

Fortification of grain products with folic acid was mandated in order to add about 0.1 mg folate to the daily intake of an adult. Some studies have shown that the resulting increase, in daily folate intake, is about 0.2 mg [10,11]. Even if a patient has no other source of folate intake, a daily intake of 0.1 mg folic acid is more than enough to prevent folate deficiency anemia. In fact, patients with folate deficiency megaloblastic anemia can be treated with as little as 0.025 mg [12] or 0.050 mg [13] of folic acid daily.

Only 11 of 2154 (0.5%) SF assayed at HSC during 2001 were subnormal. In none of these patients, was the SF as low as is seen in patients with folate deficiency anemia. Similarly, in none of the patients seen at SBGH after 1998, could the anemia or macrocytosis be attributed to folate deficiency. In the National Health and Nutrition Examination Survey (NHANES) of 1999-2000 in USA [6,7], the prevalence of low SF (<6.8 nmol/L) was 0.5% (compared to 16% in NHANES III for the period of 1988-1994) [6]. The prevalence of low SF and low RF reported by a large private Laboratory in Canada, during the period of February 1, 1999 to March 31, 2000 were 0.22% and 0.41% respectively [5]. Latif and colleagues [14] from Cleveland Clinic found 77 of 4985 (1.6%) SFs during 2001 were lower than normal. The MCV of these 77 patients was not different from those with normal SF. Only 39 of 74 patients with low SFs (0.9% of clinically suspected folate deficient patients) were actually prescribed folic acid. They commented that true folate deficiency is very rare; and questioned whether there is a role for folate determinations in clinical practice in the USA [14]. The prevalence of subnormal SF at HSC during 2001 (0.5%) was similar to prevalence of low SF in random population survey 1999-2000 of NHANES (0.5%) [6]. Joelson et al [15] reviewed the red cell folate data of 3 USA county hospitals with large cohort of indigent patients, during the years 1997, 2000 and 2004. Cutoffs for RF in these hospitals were 362 and 213 nmol/L. Using a RF cutoff 362 nmol/L, the combined incidence of folate deficiency decreased from 4.8% in 1997 to 0.6% in 2004. At a cutoff of 213 nmol/L, the incidence dropped from 0.98% to 0.09% [15]. They concluded that routine folate measurements for patients with anemia and/or macrocytosis is difficult to justify.

### Problems with Folate Assays and Normal Range

There are numerous methods for folate assays, each giving different result for assay of the same sample. Gunter et al [16] reported on interlaboratory comparison of SF and RF performed by 20 research laboratories (8 using L. Casei microbiological assays and 12 using competitive binding methods). Aliquots of six normal serum and blood were sent to each of 20 laboratories to perform duplicate assays on each sample daily for three days. There were a two to nine-fold differences in reported folate concentrations between the methods. In proficiency testing of the College of American Pathologists for folate assays [17], when aliquots of one sample of serum were sent to 1233 laboratories, using 12 different competitive binding methods of folate assays, the mean SF for the laboratories that were using the same method of folate assay ranged from 6.95 to 18.75. At SBGH where the same microbiological assay of folates had been performed since 1966, the normal RF was >225 nmol/L; and in folate deficiency megaloblastic anemias, RF was <100 nmol/L. It is for these reasons that we have commented that, in our study, in none of the subjects the anemia could be attributed to folate deficiency.

The normal range of SF and RF varies depending on the geographical location, the season during which the samples were obtained for determining the normal range and the average folate intake of the population. When SF or RF of a patient is low, it does not necessarily mean that the patient is folate deficient. It only means that the patient is consuming or absorbing less folate, compared to normal subjects in the area. It is recommended that laboratories not only report the normal range for SF and RF, but also to indicate the expected range of SF and RF for truly folate deficient individuals [16]. Most of the laboratories in US or Canada, are no longer able to provide a reference range for truly folate deficient individuals because they do not have access to sufficient number of samples of blood from truly folate deficient subjects. In the Herbert study [18], the reference range for L. Casei microbiological assay of SF was 15.9-36 nmol/L and folate deficient subjects had SF of <6.8 nmol/L.

### Relation of low folate assays and anemia due to folate deficiency

Many physicians order serum folate, B_12 _and ferritin for the investigation of anemia. If SF is low they assume that the anemia is due to folate deficiency. This is not a correct assumption. Anemia due to folate deficiency is megaloblastic. No anemia should be attributed to folate deficiency, unless there are megaloblastic changes in the peripheral blood or in the bone marrow aspirate. Even when there are megaloblastic changes in the bone marrow, the low SF or RF does not necessarily mean that the patient is folate deficient. About 2-19% of patients with pernicious anemia have low SF and, 23-62% have low RF [19]. Serum folate falls below normal within a short period of reduced dietary intake of folate. In the past, low SF was found in 9-75% of probably normoblastic hospitalized patients [19].

In the sequence of events leading to folate deficiency anemia, the fall of SF is the earliest manifestation of folate deficiency and anemia is a late manifestation of folate deficiency. When a normal man was placed on a folate deficient diet, his SF fell below normal by 3 weeks, RF fell below normal by 17 weeks, macocytic red cells appeared in the blood smear by 18 weeks and anemia and megaloblastic bone marrow developed at 19 weeks [20]. By the time that anemia had developed, his SF was 1.0 nmol/L (normal range 15.9-36 nmol/L) and his RF was 22 nmol/L.

As the result of fortification of grain products with folic acid, dietary folate deficiency has become very rare. Consequently, there is no longer any justification in ordering folate assays to evaluate the folate status of the patients. There are still some patients who may be folate deficient (very small premature babies before they are able to start taking grain products, pregnant women who are not taking multivitamins and who also avoid eating grain products, malnourished alcoholics or those with celiac disease or tropical sprue who are avoiding taking grain products). However, these patients could be easily identified by their medical history and could be placed on prophylactic folic acid therapy without testing for folate deficiency, as long as it is determined that their SB_12 _is normal.

In taking a patient's dietary history to assess dietary intake of folates, physicians should note that, unlike the past when fresh green leafy vegetables and fruits were the major source of dietary folate, now, grain products are the major source of dietary folate. In the last NHANES, bread rolls and crackers were the single largest contributor of total folate to the American diet [7].

## Conclusion

Our data show that, in the countries where grain products are fortified with folic acid, ordering serum or red cell folate for the investigation of anemia is a waste of time and resources. Low SF or RF are very rare and even in the few cases in which they are low, it is highly unlikely that they could be low enough to cause anemia. More importantly, the result of these tests is more likely to mislead the physicians than to provide any useful information.

## Competing interests

The authors declare that they have no competing interests.

## Authors' contributions

AMS conceived the research project, reviewed all the folate data and the medical record of those with low red cell folate at SBGH and wrote this manuscript. KvK reviewed all the folate data and medical record of those with low serum or red cell folate at HSC. He has read and commented on the manuscript. All authors have read and approved the final manuscript.

## References

[B1] Food and Drug Administration Food standards: amendment of standards of identity for enriched grain products to require addition of folic acid Federal Register 61 8781 8797

[B2] Health Canada Food and Drug Regulations, Amendment Schedule No. 1066. Ottawa 1998

[B3] Lawrence JM Petitti DB Watkins M Umekubo MA Trends in serum folate after food fortification Lancet 1999 354 915 6 10.1016/S0140-6736(99)03227-4 10489953

[B4] Choumenkovitch SF Jacques PF Nadeau MR Wilson PW Rosenberg IH Selhub J Folic acid fortification increases red blood cell folate concentrations in the Framingham study J Nutr 2001 131 3277 80 11739880 10.1093/jn/131.12.3277

[B5] Ray JG Vermeulen MJ Boss SC Cole DE Declining rate of folate insufficiency among adults following increased folic acid food fortification in Canada Can J Public Health 2002 93 249 53 12154524 10.1007/BF03405010 PMC6979918

[B6] Pfeiffer CM Caudill SP Gunter EW Osterloh J Sampson EJ Biochemical indicators of B vitamin status in the US population after folic acid fortification: results from the National Health and Nutrition Examination Survey 1999-2000 Am J Clin Nutr 2005 82 442 50 16087991 10.1093/ajcn.82.2.442

[B7] Dietrich M Brown CJ Block G The effect of folate fortification of cereal-grain products on blood folate status, dietary folate intake, and dietary folate sources among adult non-supplement users in the United States J Am Coll Nutr 2005 24 266 74 16093404 10.1080/07315724.2005.10719474

[B8] Waters AH Mollin DL Studies on folic acid activity of human serum J Clin Pathol 1961 14 335 344 10.1136/jcp.14.4.335 13783363 PMC480231

[B9] Hoffbrand AV Newcombe FA Mollin DL Method of assay of red cell folate activity and the value of the assay as a test for folate deficiency J Clin Pathol 1966 19 17 28 10.1136/jcp.19.1.17 5904976 PMC473152

[B10] Quinlivan EP Gregory JF Effect of food fortification on folic acid intake in the United States Am J Clin Nutr 2003 77 221 5 12499345 10.1093/ajcn/77.1.221

[B11] Choumenkovitch SF Selhub J Wilson PW Rader JI Rosenberg IH Jacques PF Folic acid intake from fortification in United States exceeds predictions J Nutr 2002 132 2792 8 12221247 10.1093/jn/132.9.2792

[B12] Sheehy TW Rubini M Perez-Santiago E Santini R Jr Haddock J The effect of "minute" and "titrated" amount of folic acid on megaloblastic anemia of tropical sprue Blood 1961 18 623 636 13911562

[B13] Zalusky R Herbert V Megaloblastic anemia in scurvey with response to 50 microgram of folic acid daily New Engl J Med 1961 265 1033 1038 14009735 10.1056/NEJM196111232652103

[B14] Latif T Hsi ED Rybicki LA Adelstein DJ Is there a role for folate determinations in current clinical practice in the USA? Clin Lab Haematol 2004 26 379 83 10.1111/j.1365-2257.2004.00649.x 15595994

[B15] Joelson DW Fiebig EW Wu AHB Diminished need for folate measurement among indigent populatios in the post folic acid supplementation era Arch Pathol Lab Med 2007 131 477 480 17516752 10.5858/2007-131-477-DNFFMA

[B16] Gunter EW Bowman BA Caudill SP Twite DB Adams MJ Sampson EJ Results of international round robin for serum and whole-blood folate Clin Chem 1996 42 1689 94 8855155

[B17] Bock JL Endres DB Elin RJ Wang E Rosenzweig B Klee GG Comparison of fresh frozen serum to traditional proficiency testing material in a College of American Pathologists survey for ferritin, folate, and vitamin B12 Arch Pathol Lab Med 2005 129 323 7 15737025 10.5858/2005-129-323-COFFST

[B18] Herbert V Aseptic addition method for Lactobacillus casei assay of folate activity in human serum J Clin Pathol 1966 19 12 6 10.1136/jcp.19.1.12 5904975 PMC473151

[B19] Chanarin I The Megaloblastic Anemias 1979 2 Blackwell Scientific Publication, Oxford 187 197

[B20] Herbert V Experimental nutritional folate deficiency in man Trans Assoc Am Physicians 1962 75 307 20 13953904

